# Challenges to the Educational “Digital Divide” in Spanish Prisons

**DOI:** 10.1007/s10610-021-09493-4

**Published:** 2021-08-07

**Authors:** Manuel Lázaro Pulido

**Affiliations:** 1 Department of Sciences of Law, School of Law, Universidad Bernardo O’Higgins, Santiago, Chile; 2 Department of Philosophy, Faculty of Philosophy, Universidad Nacional de Educación a Distancia, Madrid, Spain

**Keywords:** Spanish prison system, Education, Digital divide, Right to education

## Abstract

Education within prisons is one of the most complex scenarios in the field of education in Spain. Education is conceived in spatial and temporal coordinates that are totally alien to life in prison and often clash with economic or security and order-related contraindications that frustrate the right to education in the twenty-first century. This is an education that cannot be unconnected with digital competition, and one of its aims is to eliminate the “digital divide”. On the one hand, it has been analysed by the Spanish and European authorities that there is a need for education to respond to the challenges of today’s society, which is characterised by having moved from the analogue era to the digital era. This digital drive is designed to limit social differences. On the other hand, Spanish prison legislation guarantees the right to education, but without forgetting the special circumstances limiting rights in prisons. In Spain, the 1996 regulation does not seem to be able to respond to the existing difficulties, since its wording has become obsolete on this point. In this regard, the Council of Europe recalled different aspects which lead us to question how the right to comprehensive education should be skilfully combined with that of the restrictions specific to the prison environment. The study of these two aspects leads to the conclusion that it is a difficult challenge. The administration must therefore seek a fair balance between the public policy objectives pursued and respect for the rights of persons deprived of their liberty.

## Introduction

The issue of education in prisons is dealt with at a legal level and in the field of study of education, especially social education (Oliva, 2014). The legal approach focuses on the protection of rights, as well as procedural aspects. Educational studies focus on the practical aspects and the human-social context of the regulations. Emphasis is placed on the need for education as a means of achieving the reintegration of prisoners and improving the quality of life and personal development of prisoners.

In Spain, the *General Penitentiary Organic Law* (Jefatura del Estado, 1979), in the application of article 25.2 of the Spanish Constitution (EC), affirms the recognition of the prisoner as a subject of rights, as well as the most relevant purposes of the custody of the prisoner such as the reeducation and social reinsertion of prisoners. In this sense, it is necessary to point out that education is a fundamental human right, necessary for the exercise of other human rights and whose purpose is found in the integral development of every person and group; in short, with the right to education, we glimpse the idea of the right to have rights (Martín & Sánchez-Valverde, 2016).

The fact that the right to education in prisons is evident does not interrupt a reflection on many of its aspects (United Nations - UNESCO Institute for Educations, 1995). The educational situation in prisons is complex, but it becomes even more complicated in a society as changing as the present one. This implies that it is necessary to constantly think about both the institutional expressions where rights are exercised and their own conceptualisation. In fact, the actual conception of education itself also varies. Education in the twenty-first century has redefined its aims, its methods, its processes… Contemporary society is aware of the personal and social importance of education and has increased, quantitatively and qualitatively, the relevance of its action for every human being. This has led to an insistence on the effective realization of the right to education for personal advancement, while, by its very nature, it also constitutes a necessary tool for the rehabilitative purpose and promotion of human beings, especially those who are most vulnerable.

Maeyer (2019), points out the need to reinforce the right to education and training worldwide. This is an area in which UNESCO should play a more active leadership role, both in basic education, vocational training and lifelong learning. This feeling is shared in advanced societies and also in Spain. As stated by the European Commission’s National Support Services—Spain (NSS Spain, 2020):

“Education within prisons is one of the most complex scenarios in the field of education in our country. The average rate of the prison population in Europe is 115.7 prisoners per 100,000 inhabitants, while the Spanish rate is 32%, above the average for European countries. Thus, education in prison serves many people with little or no possibility of improving their situation. If adult education in prisons is to some degree already unknown, adult education in prisons is simply invisible”.

The objectives of education in prisons can sometimes be contradictory: to keep prisoners occupied intelligently, to inform and distract them, to reduce recidivism, to provide them with professional training, to calm them down, the pursuit of the happiness that comes with studying (Ravina-Ripoll et al., 2019) … Beyond this, education in prison is an activity aimed at personal liberation in an environment that does not allow such freedom. Education is conceived in spatial and temporal coordinates that are totally alien to space and time that one lives in prison. This circumstance causes economic, security and order-related contraindications that frustrate the right to education in the twenty-first century, an education that cannot be unconnected with digital competition and that has, as one of its aims, the elimination of the “digital divide”.

The digitisation of society and education affects every human being. And the prisoner, just because he is in prison, does not stop being a human being. The question we are asking is, can the right to education and training in prison be fully realised without taking into account the digitalisation of society and education itself? Our reflection aims to answer this question. We will look at the need for the digitisation of education within the European and Spanish agenda. Specifically, we will ask ourselves how education and the use of the Internet are present in prisons to highlight the difficulty of combining both aspects.

## Digital Criminology and Digital on Prisons

Today’s society is characterised by the transition from the analogue to the digital age. No area of the human, personal and social sphere is conceivable without the intervention of digitalisation. It is a dynamic field in itself (we are talking about the transition from Web 1.0 to Web 4.0) that has gone from computation to digitalisation, affecting not only the various orders of life but also the human concept itself. In fact, we speak of digital humanities as the place of expression of “digital culture itself in the human vital and existential environment” (Lázaro, 2020, 81). The digital society significantly affects the criminal, penal and penitentiary realm which calls “to expand substantially the traditional focus of computer and cyber-crime scholarship” (Stratton et al., 2017). In recent years, many scholars in the Anglo-Saxon field, concerned with the impact of digital technologies on crime and criminal justice, have replaced the concept of cyber-criminology in favour of a new term: digital criminology, which, we believe, responds epistemologically better to the new digital paradigm shift (from computerisation to digitalisation). This implies an extension of a technology-centred approach to a data-centred approach (Marres, 2017).

There is a sense that criminological engagement with a digital crime is underdeveloped. There is a growing awareness of the need to think about the implication of digitalisation in today’s criminology in its various aspects: digital investigation and evidence, digital justice, digital surveillance, digital engagement and digital social inequalities… (Powell et al., 2018).

There is an awareness among criminal justice actors that digitalisation affects criminal justice. We can see the influence of state-of-the-art digital tools (ICT) to collect, share, exchange and process operational data in a structured, encrypted, fully automated and interoperable way, especially in law enforcement. The presence of digitalisation in crime investigation and detection is a fact.

For its part, the justice system is making an effort to implement these developments, with examples in countries such as Japan and the Czech Republic (Záklasník & Putnová, 2019). In July 2019, the European Commission’s Directorate-General for Justice and Consumers, with the support of Eurojust and other stakeholders, launched a study to further discuss, develop and implement the concept of Digital Criminal Justice in order to realise cross-border criminal justice, the final report of which was drafted in June 2020 and published in September 2020 (European Commission—Directorate-General for Justice and Consumers, 2020).

The reality introduced by the concept of digital criminology provokes controversy about its role and implications, for example, the new challenges: empirical, methodological and ethical challenges introduced by the era of digital surveillance (Ceccato, 2019, 7). This does not preclude the fact that digitisation raises new concerns about the structure of society and how criminal justice practitioners and researchers can engage with the digital environment (Tangen, 2018). Digital criminology invites a multidisciplinary approach that encompasses criminology, psychology and behavioural sciences, philosophy of law, legal reflection and digital sciences in order to be able to respond to the legal challenges posed to the criminal field.

The prison service is no stranger to the digital reality in any of its spheres. Insofar as prisons are governed by the criteria of legality and justice, they are a key institution in modern democratic society and are permeable to its characteristics, such as the fact of the digital revolution. The digitalisation of society has affected them, not only in terms of the effective realisation of their rights, such as the right to communication or education, but also in terms of the prison environment itself and the challenges of digital criminality.

The prison system is introducing, within the measure of its limitations, digital technologies that better respond to the updating of prison services through digitisation. Its implementation is presented as an opportunity to prevent the digital divide from being a factor of social exclusion, especially when the prison user leaves this environment and reintegrates into society (Warschauer, 2004). The limitations of the implementation of digitisation in prisons come not only from the conditions of deprivation of liberty themselves but also from the structural conditions of prisons themselves.

Knight and Van De Steene (2017a) have reflected on the possibility of overcoming the structural barriers of prisons, which derive from an environment of deprivation, control and surveillance, to adapt them to the needs of the digital era defined as an open and communicative system. Their reflections on the capacity of prison services to transform these organisations through digital technology lead them to import concepts such as Smart Cities. Following this study, Victoria Knight and Steven Van De Steeneargue that digitisation changes the way rehabilitation (and punishment) is conceived. The inevitability of digital transformation in prison environments thus brings with it both light and dark features.

Although much remains to be done, steps are being taken in a number of prison systems. The Her Majesty’s Prison and Probation Service (HMPPS) invested in installing technology more widely into prisons in England and Wales to help enable delivery of the paper’s commitments, following the 2016 White Paper on Prison Safety and Reform: In-cell telephony systems, Self-service kiosks on wing landings which allow prisoners to complete administrative tasks, in-cell laptops allowing prisoners to access the same functions as through the wing self-service kiosks, Mobile devices for prison staff with access to P-NOMIS (Palmer et al., 2020, 8–9). The experience of PrisonCloud recently introduced in some newly built Belgian prisons (Knight & Van De Steene, 2017b) highlights the challenges of implementing this transition to digitisation in the prison context, including some not so positive ones such as that the use of digital technology in the cell has the potential to alter prisoner-staff interactions and the prisoner’s position of dependency, leading to new forms of isolation (Robberechts & Beyens, 2020).

Thus, the inclusion of digital technologies in a security-dominated environment poses new challenges for prison services that must not be forgotten by the end-user whose life must be governed by normalisation within the legal restriction of their rights (Van De Steene & Knight, 2017). Indeed, one cannot ignore the fact that, if there is a novel form of criminality from the digital revolution, it can also occur in the penitentiary sphere. There is always the challenge of knowing how to combine security with the exercise of rights. The Centre for Social Justice has pointed out how, although the British public is not a priori in favour of giving digital access to prisoners, some studies have shown that this perception varies widely, provided that adequate security is guaranteed and that there are verifiable results in terms of recidivism (Centre for Social Justice, 2021, 4).

Despite all the limits present in the implementation of digitisation in the prison environment, some studies have shown “that prison behavior was significantly improved and reoffending in the community was significantly reduced. Supporting information from usage data and a prisoner survey showed that the interaction with the technology produced a feeling of worth and personal control. This suggests that, by introducing prisoners to modern technology, it can transform their lives from dependency to self-responsibility, where they can learn new ways of behaving in a supportive rather than a punitive environment”. (Mcdougall et al., 2017, 478).

These limits clash with the recommendations on the education of prisoners and the recommendations of education authorities in relation to digital competences. Especially if we take into account that the Council of Europe in its second recommendation held in 1990 that “Education for prisoners should be like the education provided for similar age groups in the outside world, and the range of opportunities for prisoners should be as wide as possible”. (Council of Europe, 1990). This is possible if we develop the indications on digital competences from the different educational authorities.

## Education on the Digital Agenda for Europe

The European Commission is aware of the importance of digitisation and therefore publishes the *Communication from the Commission to the European Parliament, the Council, the European Economic and Social Committee and the Committee of the Regions A Digital Agenda for Europe* (2010), one of the seven pillars of the Europe 2020 Strategy which sets objectives for the growth of the European Union (EU) by 2020. “The Digital Agenda proposes to better exploit the potential of Information and Communication Technologies (ICTs) in order to foster innovation, economic growth and progress”. Digitalisation affects the very structure of the Spanish Government, which is aware of the need to implement this reality in the social and economic tissue. This reality is a commitment to education, since twenty-first-century education is digital as well as all of its educational actors. Thus, in June 2020, the Council of Ministers approved the signing of an agreement among the Ministry of Education and Professional Training, the Ministry of Economic Affairs and Digital Transformation, and Red.es to launch the “Educa en Digital” (“Educate in Digital”) programme to support the digital transformation of education in Spain. Thus, the *Resolution of 7 July 2020, of the Subsecretary for the execution of the “Educate in Digital” programme* (Ministerio de la Presidencia, Relaciones con las Cortes y Memoria Democrática, 2020), states in its Annex 1 that: “The process of the digital transformation of education in Spain requires the generalisation of the use of online resources, telematic tools for communication and collaboration and Internet devices and connections by both teaching staff and students, and is demanding intensive use of ICT, both in the classroom and in non-presential formats”.

The actions are expected to start during the first quarter of the 2020–2021 academic year. The programme does not focus so much on making education digital, but rather on guaranteeing the right to a twenty-first century education. This means providing digital education to the most vulnerable students. To this end, the centres are provided with a budget so that they have devices that facilitate digital education both in the centre and from the home. This action is not only limited to Web 2.0 tools but also affects the digital devices of the Web 4.0 network. The programme establishes the implementation of assistance platforms for teachers, students and educational authorities through the application of Artificial Intelligence to promote a more personalised education. This development will make it possible to establish personalised itineraries for students, more effective monitoring of their progress and an individualised analysis of their progress by teachers.

Further digitisation is not a question of mere modernisation but is deeply embedded in the current learning process, as indicated in the cited Agreement (Ministerio de la Presidencia, Relaciones con las Cortes y Memoria Democrática, 2020) in no. 8 of the Annex.

The Ministry underlines the new educational reality, the key of which is no longer based only on content, but fundamentally on competences. It follows the indications generally presented in the educational literature and endorsed by the European educational authorities. The Committee of the Regions (2006) highlighted this reality when they recommended that the Member States “develop the provision of key competences for all as part of their lifelong learning strategies”.

In this document, the EU defined the key competences as a combination of “knowledge, skills and attitudes that lead people to be more involved in both sustainable development and democratic citizenship”. They are all considered to be of equal importance since they contribute equally to success in the knowledge society. The European Parliament ratified its position (2006), and it defines digital competence: “Digital competence involves the confident and critical use of Information Society Technology (IST) for work, leisure and communication. It is underpinned by basic skills in ICT: the use of computers to retrieve, assess, store, produce, present and exchange information, and to communicate and participate in collaborative networks via the Internet”. It is impossible to achieve an adequate development of this competence without its study, understanding and application.

On the other hand, it is clear that the development of individual competences and the dissemination of knowledge and skills are substantial in the development of an inclusive society, as they are one of the key elements of integrated employment and social policies and can enable long-term growth to be generated, European competitiveness to be enhanced, unemployment to be combated and a more inclusive European society to be built. Of these, digital competence is currently the most important (European Commission, 2020). The importance given to this digital competence or capacity is such that the European Commission states its desire to “Develop a European Digital Skills Certificate (EDSC)”. This commitment implies that the States must guarantee free access to this training since if this does not happen, there would be discrimination due to digital competition, which would affect both the development of the person and his or her right to social promotion and labour insertion.

We can say that a full education cannot be understood without the full development of digital competence. This is set out in the different regulations of Spanish legislation. The digital competence is underlined at different levels of education. *Royal Decree (RD) 1513/2006, which establishes the minimum teaching requirements for primary education* (Ministerio de Educación y Ciencia, 2006), underlines digital competence (Annex I) and for their part, in the RD 1105/2014, *establishing the basic curriculum for compulsory secondary education and the baccalaureate* (Ministerio de Educación, Cultura y Deporte, 2015), the development of digital competence is similar to that established in Primary Education.

Despite the complexity of education in prisons, it is clear that “offering disadvantaged students a second chance to learn, finding ways to recognise and validate knowledge, taking advantage of the use of new technologies and expanding their opportunities, acts to the benefit of all, i.e., it is not just a one-way process of reducing recidivism. Statistics show that low skill levels have a significant negative effect on the employability of prisoners after release and are one of the main reasons why prisoners reoffend”. (NSS Spain, 2020) One of the priorities is to guarantee the right to education, bearing in mind that the important thing is “Education for Freedom”. In this respect, it should not be forgotten that the prisoner who is trained in prison is still a student. The student prisoner resists being taken as an inmate in the prison; he cannot be fully captured as an inmate in the prison in which he is imprisoned. The student prisoner, though imprisoned, is not only a prisoner but a human being in prison and therefore a student as well. In this process of individual progress, digital literacy helps the subject to build a digital identity as an autonomous, educated and democratic citizen on the Net. This objective is especially desirable for those who are imprisoned (Area, & Pessoa, 2012). We cannot forget that education in prisons is part of a continuum involving rights, freedom and citizenship (UNESCO, 2008). It is difficult to understand an educational development without digital immersion. The provision of material and personal resources to carry them out and the establishment of incentives for the participation of inmates is a requirement to comply with the fundamental right to education in Spanish legislation (art. 27 CE) in the context of deprivation of liberty (Rodríguez, 2013).

There is no doubt that the digital world, the ICTs—information and communication technologies (Internet…)—are understood as a means of improving and promoting people’s autonomy and as a participatory element that favours social cohesion, intrinsic to the effective right to education and positive in terms of favouring the reinsertion of vulnerable people, among them, especially those in prisons.

The importance of digital devices, especially the use of the Internet, has been recognised in various international regulations. The Council of Europe (2003) points out the importance of bridging the digital divide. *The Directive (EU) 2016/2102* on the accessibility of the websites and mobile applications of public sector bodies (European Parliament and the Council of the European Union, 2016), recalls the need for the universalisation (“universal design”) of access to digital resources, which is particularly sensitive to vulnerable groups. For its part, Recommendation CM/Rec(2014)6 of the Committee of Ministers to member States on a Guide to Human Rights for Internet users was adopted on 16 April 2014 (Council of Europe—Committee of Ministers 2014), indicating the importance of universal access and non-discrimination.

The doctrine on this subject by the United Nations (UN) is clear, as it recalls the Human Rights Council (2018) at its Thirty-eighth session. We recall the statements made by the Special Rapporteur of the United Nations Human Rights Council on the promotion and protection of the right to freedom of opinion and expression, Frank La Rue, in his report of 16 May 2011 to the Human Rights Council – United Nations (2011): “61. The term “digital divide” refers to the gap between people with effective access to digital and information technologies, in particular the Internet, and those with very limited or no access at all. In contrast to 71.6 Internet users per 100 inhabitants in developed States, there are only 21.1 Internet users per 100 inhabitants in developing States”.

Nobody doubts the need in the twenty-first century to promote the use of digital tools to all human beings in order to avoid the “digital divide” as a basic element to guarantee equal opportunities. Some authors in the vortex of the extension of human rights—in a position that is becoming commonplace and possibly in what constitutes the greatest attack on the referential essence of fundamental rights themselves and their positivity—go so far as to speak of “digital inclusion” as a new human (López, & Samek, 2009) or fundamental right (Chacón-Penagos et al., 2017). This may be a conceptual outrage, but it shows the importance of digital reality as a basic tool in the implementation of human and fundamental rights in the twenty-first century.

In short, the binomial “education and digital” can no longer be dissociated, the digital reality being a transversal instrument to the different rights, including especially the right to education. Education in the twenty-first century cannot be understood without the digital reality and, therefore, it is necessary to include digital education as one of the fundamental elements for the effective realisation of the right to education: “4. Affirms that quality education plays a decisive role in development, and therefore calls upon all States to promote digital literacy and to facilitate access to information on the Internet for all children, which can be an important tool in facilitating the promotion of the right to education, and to support similar learning modules outside of schools”.

## Digitisation and Prisons Education

Faced with this new reality, we cannot forget the special circumstances of limitation of rights existing in prisons, due to their nature, especially with regard to the right of communications and the use of electronic and digital devices. The R.D. 190/1996, which approves the Prison Regulations (Ministerio de Justicia e Interior, 1996) establishes in its article 129, regarding the “Disposal of personal computers”:

“1. When educational or cultural reasons make it necessary or advisable for the development of the corresponding training programmes, the intern may be authorised to have a personal computer. To this end, the inmate will be required to present a report justifying the need, endorsed by the Teacher or Tutor. 2. The use of the computer and the computer material will be regulated in the corresponding internal rules and, in any case, the transmission of tapes or “diskettes” and the connection to communication networks will be forbidden. 3. The Board of Directors may withdraw the authorization granted when there are reasonable suspicions that it is being misused or when the authorization does not correspond to a real need of the intern. In any case, it shall be understood that there is a suspicion of misuse of the computer when the intern refuses to show the content of all the files of the computer, after having been requested to do so by the Governing Board”.

It should be noted that this is a 1996 regulation. The legislation does not even refer to the term “Internet”, let alone the concept of “digital”. We are talking about a time in which we are in the first stage of digital evolution, a time which is perhaps only computational. We are talking about the Web 1.0 which is defined by its unidirectionality, the webmaster exerting absolute control over the content in an environment defined by the static pages. The emphasis of Web 1.0 was to “publish” content on the Web for users, not content provided by the user. This was a time when browsers were unsophisticated and had limited access to the Internet, often through analogue speeds of 56 K or less (Murugan et al., 2010). This was an era in which education had not yet looked to ICTs, let alone digitalisation as a reality already inherent in that activity. These restrictions were logical in the culture of prison, based on its characteristics dominated by security. However, it is difficult to be consistent with what is currently stated in Article 130 on Professional and Occupational Training (Ministerio de Justicia e Interior, 1996), which states: “2. the courses will be organised in accordance with existing plans for other citizens in the field of vocational and occupational training and social and labour integration. 3. vocational training shall consist of the theoretical and practical parts set out in the corresponding plans”.

Or with the provisions of Article 126 relating to Educational Units: “3. The educational facilities will be conditioned and will have the necessary material means to carry out the training activities under the control of the Educational Unit”.

It is unlikely that digital deficits will contribute to the provisions of Article 124 when it states that: “1. The Penitentiary Administration shall facilitate the access of inmates to regulated and non-regulated educational programmes that contribute to their personal development”.

In fact, the orientation of prison sentences in the Spanish prison model is re-education and social reintegration. This objective implies opening up to the outside world in order to prepare prisoners for their future life in society. This objective cannot be achieved at present without the inclusion of the digital reality, unless education is encouraged to deepen the “digital divide”, which contradicts the above principles. In 2017, a Spanish deputy asked the Ministry of the Interior about the lack of broadband Internet in prison. This absence prevented prisoners from accessing distance learning high school studies (Europa Press—Andalucía, 2020). The MEP highlighted the need to address the unavoidable issue of the relevance of ICTs and digital reality as a standardised form of current social life and education in particular.

Restricted access means limitations on education and culture, basic and advanced training, employment, communication with the outside world, treatment, or even leisure and entertainment. Its limitation means restricting it to the paths of humanisation of the twenty-first century (Fernández, 2018). The deprivation of liberty should not nullify or limit other rights of individuals who are in prison, who remain persons in law (Franganillo et al., 2006).

The Council of Europe recalled in *Recommendation Rec(2006)2* of the Committee of Ministers to member States on the European Prison Rules, different aspects that lead us to question the need to skilfully combine the right to a comprehensive education with that of prison-specific restrictions (Council of Europe – Committee of Ministers, 2020b). In no. 24.2, it was noted that “Communication and visits may be subject to restrictions and monitoring necessary for the requirements of continuing criminal investigations, maintenance of good order, safety and security, prevention of criminal offences and protection of victims of crime, but such restrictions, including specific restrictions ordered by a judicial authority, shall nevertheless allow an acceptable minimum level of contact”. This principle should be articulated with regard to an education that provides “Every prison shall seek to provide all prisoners with access to educational programmes which are as comprehensive as possible and which meet their individual needs while taking into account their aspirations” (28.1). The recommendation states that where there is a “library for the use of all prisoners”, this library is “adequately stocked with a wide range of both recreational and educational resources, books and other media”. (28.5). The aim is to ensure that, as far as possible, the education of prisoners is integrated into the country’s educational and professional training system. “As far as practicable, the education of prisoners shall: a. be integrated with the educational and vocational training system of the country so that after their release they may continue their education and vocational training without difficulty; and b. take place under the auspices of external educational institutions”. (28.7).

The problem arises from the difficulty of using digital tools. But that difficulty does not prevent the possibility of their implementation. There are experiences of their supervised use in prisons, so their use is not a chimera.

In fact, the Appendix to Recommendation CM/Rec (2018) 5 of the Committee of Ministers to member States concerning children with imprisoned parents (Council of Europe—Committee of Ministers, 2020a). encourages their use:

“In accordance with national law and practice, the use of information and communication technology (video-conferencing, mobile and other telephone systems, Internet, including webcam and chat functions, etc.) shall be facilitated between face-to-face visits and should not involve excessive costs”.

The rules in the appendix continue the path of increasing sensitivity regarding the place of new information technologies and were relatively upheld by the Court in Strasbourg (Turgis, 2013). The European Court of Human Rights-ECHR, half-opened Internet access for prisoners in his sentence of 19 January 2016*: Case Kalda v. Estonia* (ECHR, 2016). Although Article 10 on freedom of expression of the *Convention for the Protection of Human Rights and Fundamental Freedoms* (ECHR – Council of Europe, 2013) cannot be considered the source of a real right of access to the Internet for prisoners, the judges in Strasbourg nevertheless require that any interference with the right of prisoners to receive information by this means be “provided for by law”.

This sentence (*Case Kalda v. Estonia*) has become final under Article 44 § 2 of the Convention. It may be subject to editorial review.

The case originates from Romeo Kalda’s application (No. 17429/10) to the European Court of Human Rights (ECHR) against the Republic of Estonia on 16 March 2010. The applicant claimed that his right under article 10 of the Convention to receive information via the Internet without interference by a public authority had been violated (art. 10.1).

Let us recall that Article 10 of the ECHR regulates freedom of expression:

“1. Everyone has the right to freedom of expression. This right shall include freedom to hold opinions and to receive and impart information and ideas without interference by public authority and regardless of frontiers. This Article shall not prevent States from requiring the licensing of broadcasting, television or cinema enterprises. 2. The exercise of these freedoms, since it carries with it duties and responsibilities, may be subject to such formalities, conditions, restrictions or penalties as are prescribed by law and are necessary in a democratic society, in the interests of national security, territorial integrity or public safety, for the prevention of disorder or crime, for the protection of health or morals, for the protection of the reputation or rights of others, for preventing the disclosure of information received in confidence, or for maintaining the authority and impartiality of the judiciary”.

The issue at stake relates to restrictions on access by prisoners in prisons to certain websites containing legal information.

The applicant, Mr Romeo Kalda, is a prisoner whose complaint is that he was prevented from carrying out a legal investigation as a result of being refused access to certain Internet sites. These included the website of the local Council of Europe Information Office and some, but not all, state databases containing legislation and court decisions.

In the appeal proceedings initiated by the plaintiff, the Supreme Court concluded that granting access to Internet sites beyond those authorised by the prison authorities could *increase the risk of prisoners engaging in prohibited communications*, thus necessitating greater control over their use of computers.

The demand revolves around Article 10 of the ECHR. What is at issue is not the refusal of the authorities to provide the requested information. Rather, the complainant’s complaint concerned a specific means of accessing—namely via the Internet—information published on certain websites that were freely available in the public domain.

The conditions attached to prisoner status—imprisonment—inevitably entailed several restrictions on prisoners’ communications with the outside world, including their ability to receive information. Article 10 could not be interpreted as imposing a general obligation on prisoners to provide them with access to the Internet, or to specific Internet sites. However, in the circumstances of the case, since prisoners were granted limited access to the Internet under domestic law—including access to official databases of legislation and court decisions—restricting access to other sites that also contained legal information had constituted an interference with the right of the applicant to receive information. The interference was prescribed by law and pursued the legitimate objectives of protecting the rights of others and preventing disorder and crime:

“In this connection, the Court reiterates that in the light of its accessibility and its capacity to store and communicate vast amounts of information, the Internet plays an important role in enhancing the public’s access to news and facilitating the dissemination of information in general (see Delfi AS v. Estonia [GC], no. 64569/09, § 133, ECHR 2015; Ahmet Yıldırım v. Turkey, no. 3111/10, § 48, ECHR 2012; and Times Newspapers Ltd v. the United Kingdom (nos. 1 and 2), nos. 3002/03 and 23676/03, § 27, ECHR 2009).

§45. Nevertheless, the Court notes that imprisonment inevitably involves a number of restrictions on prisoners’ communications with the outside world, including on their ability to receive information. It considers that Article 10 cannot be interpreted as imposing a general obligation to provide access to the Internet, or to specific Internet sites, for prisoners. However, it finds that in the circumstances of the case, since access to certain sites containing legal information is granted under Estonian law, the restriction of access to other sites that also contain legal information constitutes an interference with the right to receive information”.

The websites to which the applicant had requested access contained predominantly legal information and information related to fundamental rights, including the rights of prisoners. The accessibility of such information promoted public awareness and respect for human rights. Such information was used by national courts and therefore the applicant also needed access to it for the protection of his or her rights in legal proceedings. When the applicant filed his complaint with the national courts, the Estonian language translations of the European Court’s judgments against the respondent State were only available on the website of the local Council of Europe office to which access had been refused.

Several Council of Europe and international legal instruments had increasingly understood access to the Internet as a right and had called for effective policies to achieve universal access to the Internet and overcome the “digital divide”. Also, more and more services and information were being made available only on the Internet.

Finally, under domestic law, prisoners have been granted limited access to the Internet employing specially adapted computers and under the supervision of the prison authorities. The necessary arrangements have therefore already been made for the use of the Internet by prisoners and the authorities have already covered the costs involved. The national courts have not taken due account of the possible security risks arising from the use by the complainant of the websites in question, bearing in mind that these are administered by the Council of Europe and by the State itself. Nor had it been demonstrated that giving the complainant access to three additional websites would have resulted in any significant additional costs. In short, while the security and economic considerations referred to by the national authorities could be considered relevant, they had not been sufficient to justify interference with the applicant’s right to receive information.

The court’s conclusion (by six votes to one) is that there that there has been a violation of Article 10 of the Convention. By virtue of the judgment, the Committee of Ministers (ECHR, 2019) adopt the Resolution CM/ResDH (2019)109, of execution of the judgment of the European Court of Human Rights Kalda against Estonia. The Committee of Ministers recalls “the respondent State’s obligation, under Article 46, paragraph 1, of the Convention, to abide by all final judgments in cases to which it has been a party and that this obligation entails, over and above the payment of any sums awarded by the Court, the adoption by the authorities of the respondent State, where required: of individual measures to put an end to violations established and erase their consequences so as to achieve as far as possible restitutio in integrum; and of general measures preventing similar violations”.

The Committee of Ministers follows the ECHR’s guidance that states are not obliged to provide detainees with access to the Internet. However, if a Contracting State agrees to allow such access, it must give reasons for refusing to provide access to certain sites. In the specific circumstances of the case, the reasons given for refusing the applicant access to the three Internet sites in question (namely security reasons and cost considerations) were not sufficient to justify interference with the applicant’s right to receive information.

What the ECtHR suggests doctrinally with this ruling is that it is necessary to provide for other possibilities of access to useful information. In particular, it is necessary to consider access to education and training for this purpose. In this way, access to the Internet is progressively and inevitably understood as a fundamental tool for the development of rights, since proposing an alternative is not viable, but rather it would fall into the “digital divide”.

For its part in the judgment *Case Jankovskis v. Lit*huania (17 January 2017) (ECHR, 2017), the ECHR follows the previous judgment (*Kalda v. Estonie*, §§ 23–25) in favour of the applicant. The applicant was a prisoner, who complained that he had been refused access to a website run by the Ministry of Education and Science, which would have prevented him from obtaining educational information, in violation of Article 10 of the ECHR. The point at issue is not the authorities’ refusal to provide the requested information, but rather the applicant’s complaint concerns a particular means of access—the Internet—to information in the public domain and published on a freely accessible website. He needs access to a platform (AIKOS) in order to continue his university studies. Access was refused. The ECHR argues: “Turning to the Lithuanian authorities’ decisions, the Court cannot but observe that they essentially focussed on the legal ban on prisoners having Internet access as such, instead of examining the applicant’s argument that access to a particular website was necessary for his education”. (§ 61).

The Court continues to state in an enlightening way that: “Lastly, the Court is mindful of the fact that in a number of the Council of Europe’s and other international instruments the public-service value of the Internet and its importance for the enjoyment of a range of human rights has been recognised. Internet access has increasingly been understood as a right, and calls have been made to develop effective policies to achieve universal access to the Internet and to overcome the “digital divide” (see Kalda, cited above, § 52)”. (§ 62).

The ECUR “is not persuaded that sufficient reasons have been put forward in the present case to justify the interference with the applicant’s right to receive information” (§ 63) and decides in favour of the applicant.

The recommendations of the ECHR insist on the development of policy measures that are effective in overcoming the aforementioned “digital divide”. Measures must be taken to combine the effective right to education with the necessary security protection in order to avoid indiscrimination which would be contrary to the very purpose of this type of structure, which is to provide education and support for social reintegration. The ECHR underlines the need for legal provision for the new circumstances introduced by the digital culture and reality. Can a 1996 regulation respond to the new needs and realities that cannot be ignored for the effective and real exercise of the right to education in the twenty-first century? The ECHR (1978) recalls that “the Convention is a living instrument which, as the Commission rightly stressed, must be interpreted in the light of present-day conditions” (*Case Tyrer v. United Kingdom*, 25 April 1978), § 31).

The introduction of the COVID-19 pandemic into the lives of men has made society aware of the importance of the use of digital platforms in the effective realisation of certain fundamental rights such as that of communications and has accelerated the implementation of communication security devices. This has led to a shift in the perspective of the relevance of their use in the penitentiary environment, especially as the epidemic in Spain has led Penitentiary Institutions to suspend prisoners’ leave and visits from the outside to prisons (Güerri et al., 2021), in order to try to prevent the virus from spreading through the penitentiary centres, in line with WHO guidance of 15 March 2020 (WHO/Europe, 2020). The spread of the COVID-19 pandemic must incorporate humanitarian measures (Rodríguez, 2020). For this reason, in order to facilitate prisoners’ contacts with their families, call time has been extended and the use of mobile phones and special booths for video calls has been introduced. However, the modification of the use is not sufficient in the face of the rigidity of the regulation as it appears in its Art. 41. (Ministerio de Justicia e Interior 1996). And in any case, this use especially addresses the issue of communications, especially with regard to video calls, which appears as an increasingly unavoidable reality in special circumstances such as communication with the lawyer (García, 2019) at a broader level (García, 2020).

The problem of the dichotomy between security and rights is closely linked in legislation and case law to that of the relationship between the disciplinary regime for prisoners,^1^ which “is aimed at ensuring security and good order in the regime. It aims at an orderly coexistence,” and that of prison treatment which “has an exclusively resocialising purpose and requires multiple activities which are modulated in a long and complex process of training”. The two principles do not always coincide, but they are complementary (Muñoz, 2019), being implemented as an essential aspect in the Treatment Area as a goal of the system (Montero & Buron, 2020), although the fundamental circumstances of penitentiary treatment must always be taken into account, as the Judgment of the Criminal Chamber of the Spanish Supreme Court points out. The court thus establishes a prevalence of the reintegrative interpretation of the inmate in its context (STS FJ.3, Tribunal Supremo, 2019). Indeed, Instruction 3/10 on *Protocol of action in security matters states* that it is necessary to follow the “experience that advises the division of security regulations, separating, therefore, all instructions relating to the monitoring and control of inmates belonging to terrorist organisations, national or international organised crime and those whose violence and criminal capacity has led them to commit very serious crimes, from those of a general nature”. (Ministerio del Interior – Secretaría General de Instituciones Penitenciarias, 2010, 2).

In this sense, it seems that for all of the above reasons, Spanish legislation must reformulate the implementation of Annex II of the catalogue of objects prohibited in the penitentiary regime, with regard to electronic and computer equipment (Ministerio del Interior – Secretaría General de Instituciones Penitenciarias, 2010, 57). The jurisprudence tends to allow the use of elements that favour the effective carrying out of studies, provided that they are contained in the said Annex II [Auto de Audiencia Nacional Sala penal, Sección 1ª de fecha 03/12/18, in which the appeal on authorisation to keep a lamp in the cell was dismissed, FJ 1] (Audiencia Nacional, 2019).

## Conclusion

In the case we are analysing, the implementation of digitalisation in education in prisons not only produces the paradigmatic clash between freedom and security, but also creates a real obstacle to the effective realisation of fundamental rights, which can be retained, but not annulled.

The solution is not easy. But just as there are experiences of using web technologies and digital resources in prisons in activities such as libraries or communications, measures should be taken to facilitate the use of these new tools in education to avoid a “digital divide”. This “divide” is counterproductive to the objectives that encourage education in prisons and the effective development of this right. Indeed, if security measures have already been adopted concerning the use of the Internet by prisoners using specially adapted computers and under the control of the prison authorities, thought should be given to why serious consideration should not be given to this issue. In fact, what the ECHR suggests is that national courts have not carried out any detailed analysis of the security risks that could arise from allowing access to the additional sites in question.

Aware of this, the French authorities are taking a step in the direction of case-law by anticipating the problem, in the knowledge that access to the internet is essential for the respect of fundamental rights and freedoms, hence they claim that: “Although the case law of the European Court of Human Rights has so far not enshrined a fundamental right to access the Internet, in particular in its decisions concerning prisons (4), the fact is that the Internet has become, over time and in a particularly sensitive manner in places of deprivation of liberty, an indispensable means of ensuring the effective exercise of many fundamental rights”. (Contrôleur général des lieux de privation de liberté, 2019). The administration must therefore seek “a fair balance between the public policy objectives pursued and respect for the rights of persons deprived of their liberty, who, although not free to come and go to assert their rights personally, are destined to regain their freedom of movement in the short or long term” (Contrôleur général des lieuxde privation de liberté, 2019).

Today, the effective exercise of the right to education (primary, secondary, high school, vocational training, university, lifelong learning…) cannot be understood without access to the appropriate tools, i.e. a sufficient number of terminals connected to the Internet, in places of deprivation of liberty, to be able to fully carry out their work. Nor is it conceivable, as the French authorities point out, that “persons deprived of their liberty who wish to benefit autonomously from computer, Internet or other distance learning training, or who need to seek and consult information online, must also have access to suitable connected equipment” (Contrôleur général des lieux de privation de liberté, 2019).

As we have pointed out, the COVID-19 experience has raised the unavoidable presence of new challenges in Prisons (Solar & Lacal, 2020), including significantly that of digital media in human life. According to the press (A.R.E., 2021), this reality is prompting the Ministry of the Interior to consider amending the Prison Regime Regulations. This amendment would affect the nature of communications (art. 41) and also the updating of article 129 on “Provision of personal computers”, opening the door for the internal rules to regulate the use of computers and computer equipment, “including the use of external information storage devices and connection to communication networks”. It would also include making art. 127 on “Libraries” more flexible so that they can have access points to information networks, in accordance with the principles in force at any given time in terms of digital security and data protection. As the press release points out, the Ministry intends to indicate that “the use of these media, both for the purposes set out in article 128 and in general in the educational or cultural sphere, will be regulated by the internal rules of each penitentiary Centre, and limitations may be established individually under the terms set out in article 128” (A.R.E., 2021). The mere presence of the news item indicates the urgency of responding to this question. The issue of digitisation is a fact that deserves to be legislated. To our knowledge, there is hardly any case law on the subject. The judicial bodies must be provided with clear tools of interpretation so that the maxim that *in claris non fit intepretatio* can be applied.

An example of anticipation can be found in the document *Líneas estratégicas del modelo de ejecución penal en Navarra* in which the Government of Navarre has proposed to “Improve access to computer and digital education resources” with the aim that inmates can “reach the same training offer as on the outside, so as to guarantee this equality in the right to education”. (Gobierno de Navarra, 2021, 67, 68).

The National University of Distance Education, which is responsible for university education in prisons, is a pioneering example in the implementation of digital platforms with platforms such as Alf that are still not yet being used by inmates in a standardised way. Other platforms are being implemented, however, slowly, under the awareness that as Carlos Fernández said in 2020, “New developments in the prison context, are enhancing and broadening educational possibilities for inmates; such technological developments, focused originally in security issues, have also proved their effectiveness when implemented on educational issues, No threats to security have been reported so far, and these developments are promoting chances for further re-integration into society, as they improve personal skills on the whole”.

The challenge is not easy, that is why it is a challenge, but it is unequivocal and it is inevitable. Regulations, jurisprudence, educational literature, the actions of our neighbouring countries… must encourage us to take seriously the effective realisation of the right to education and the overcoming of the “digital divide” in prisons.

## Data Availability

Not applicable.

## Footnotes

1 The philosophical problem underlying the dichotomy between freedom and security is profound and would involve an article in itself. Paradigmatically raised in Hobbes, but discussed before, especially since the creation of the concept of the person and legal subject in the Middle Ages, the solutions have been balanced among those who are in favour of security, those who are in favour of freedom and those who consider this to be a false controversy. As premises, we recall what the United Nations has said in the Manual on Human Rights Training for Prison Officials: “‘Human rights’ is a modern term but the principle that it invokes is as old as humanity. It is that certain rights and freedoms are fundamental to human existence. They are inherent entitlements that come to every person as a consequence of being human, and are founded on respect for the dignity and worth of each person. They are not privileges, nor gifts given at the whim of a ruler or a Government. Nor can they be taken away by any arbitrary power. They cannot be denied, nor can they be forfeited because an individual has committed any offence or broken any law.” (United Nations 2005, 4).
